# Patient-reported outcomes and experiences of migrants enrolled in a multidisciplinary HIV clinic with rapid, free, and onsite treatment dispensation: the ‘ASAP’ study

**DOI:** 10.1186/s12981-024-00632-5

**Published:** 2024-06-18

**Authors:** Anish K. Arora, Serge Vicente, Kim Engler, David Lessard, Edmundo Huerta, Joel Ishak, Nadine Kronfli, Jean-Pierre Routy, Joseph Cox, Benoit Lemire, Marina Klein, Alexandra de Pokomandy, Lina Del Balso, Giada Sebastiani, Isabelle Vedel, Amélie Quesnel-Vallée, Bertrand Lebouché, ASAP Migrant Advisory Committee

**Affiliations:** 1 Department of Family Medicine, Faculty of Medicine and Health Sciences, McGill University, Montréal, Québec Canada; 2 Centre for Outcomes Research & Evaluation, Research Institute of the McGill University Health Centre, 5252 de Maisonneuve Blvd - Office 3C.25, Montreal, QC H4A 3S5 Canada; 3 Infectious Diseases and Immunity in Global Health Program, Research Institute of the McGill University Health Centre, Montréal, QC Canada; 4 Canadian Institutes of Health Research Strategy for Patient-Oriented Research (CIHR/SPOR) Mentorship Chair in Innovative Clinical Trials in HIV Care, Montréal, Canada; 5 Department of Medicine, Chronic Viral Illness Service, Division of Infectious Diseases, Department of Medicine, McGill University Health Centre, Montréal, QC Canada; 6 Department of Epidemiology, Biostatistics and Occupational Health, Faculty of Medicine & Health Sciences, McGill University, Montréal, QC Canada; 7 Pharmacy Department, McGill University Health Centre, Montréal, QC Canada; 8 Lady Davis Institute, Jewish General Hospital, Montréal, QC Canada; 9 Department of Sociology, Faculty of Arts, McGill University, Montréal, QC Canada

**Keywords:** Migrants, HIV, Patient-reported outcomes, Care experience, Multidisciplinary

## Abstract

**Background:**

Scholars recommend providing migrants living with HIV (MLWH) with free treatment, rapidly, once linked to care to optimize their HIV-related experiences and health outcomes. Quantitative evaluations of patient-reported measures for MLWH in such models are necessary to explore the viability of these recommendations.

**Methods:**

Within a 96-week prospective cohort study at a multidisciplinary HIV clinic, participants received bictegravir/emtricitabine/tenofovir alafenamide (B/F/TAF) for free and rapidly following care linkage. Eight patient-reported measures were administered at weeks 4, 24, and 48: (1) mMOS-SS to measure perceived social support; (2) IA-RSS to measure internalized stigma; (3) K6 to measure psychological distress; (4) PROMIS to measure self-efficacy with treatment taking; (5) G-MISS to measure perceived compliance with clinicians’ treatment plans; (6) HIVTSQ to measure treatment satisfaction; (7) CARE to measure perceived provider empathy; and (8) PRPCC to measure perceived clinician cultural competence. Linear mixed modelling with bootstrapping was conducted to identify significant differences by sociodemographics and time.

**Results:**

Across weeks 4, 24, and 48, results suggest that MLWH enrolled in this study experienced moderate levels of social support; elevated levels of HIV-related stigma; moderate levels of distress; high self-efficacy with daily medication self-management; great compliance with clinicians’ treatment plans; high treatment satisfaction; high perceived empathy; and high perceived cultural competence. Experience of social support (i.e., mMOS-SS scores) differed significantly by birth region. Experience of HIV-related stigma (i.e., IA-RSS scores) differed significantly by birth region, age, and language. Experience of distress (i.e., K6 scores) differed significantly by sexual orientation. Experience of treatment satisfaction (i.e., HIVTSQ scores) differed significantly by birth region and age. No significant differences were identified by time for any measure.

**Conclusion:**

Overall, participants expressed positive experiences around treatment and care, alongside comparably lower perceptions of social support, internalized stigma, and distress, potentially underscoring a need to embed targeted, well-funded, and accessible mental health support within HIV care models.

## Introduction

The HIV field has been a champion in progressing global thought and action towards developing models of care that focus on the lived experiences, needs, and preferences of people and populations [1]. This, in turn, has encouraged the design and implementation of patient-centered health systems for people living with HIV (PLWH) [1–3] which: advance a holistic understanding of HIV and the multifaceted challenges PLWH present with; reorient the focus of HIV care and research efforts to go beyond simple survival, and instead strive to ensure that PLWH also thrive in their lives; and ultimately, to develop, scale-up, and optimize models of care which allow for sustained and meaningful engagement [1, 4, 5]. Alongside adopting patient-centric approaches, HIV scholars have called for an equity-focused approach to ending the HIV epidemic, whereby efforts are systematically targeted to specific populations with the heaviest burden of HIV [6, 7].

People who relocate temporarily or permanently across international borders for any reason (henceforth ‘migrants’), particularly to member countries of the Organization for Economic Co-Operation and Development (OECD), require specific considerations from HIV specialists [8]. Migrants experience a high burden of HIV and account for a large proportion of new HIV incidence across OECD countries [9–12]. For example, in 2020, 44% of those diagnosed with HIV in Europe were migrants, many of which are suggested to have acquired HIV after arrival in the European Union / European Economic Area [12]. Similarly, in 2020, migrants accounted for 45% of new HIV diagnoses in Canada, of which 54% were diagnosed after their arrival [13]. Migrants living with HIV (MLWH) encounter numerous intersectional barriers which hinder their access to and engagement with HIV care [8, 14]. For instance, MLWH can experience lack of secure and sufficient housing, food, income, legal status, social networks, knowledge around health system navigation, language proficiency, and mental health support [8]. Additionally, experiences and perceptions of stigma based on one’s HIV and migrant statuses can potentially intersect and amplify the perceived vulnerability of MLWH, further hindering their engagement with HIV care and treatment [8].

To potentially alleviate challenges faced by MLWH at the clinical level, and thereby improve HIV-related health outcomes, previous work with MLWH suggests the importance of providing migrants with free antiretroviral therapy (ART) dispensed on-site, as well as free-of-charge HIV care (i.e., cost-covered blood tests and clinician visits), as soon as possible after HIV diagnosis [8]. This combination of factors can enable efficient access to care and treatment, particularly for migrant populations who may have just arrived in their host country, may not have immediate access to public health insurance, and may be unfamiliar with their new local health system [15]. Furthermore, rapid ART initiation has been shown to reduce loss-to-follow-up between HIV testing and treatment initiation, improve retention in care, and reduce time to HIV viral suppression, without compromising safety [15]. Additionally, previous research with MLWH suggests the importance of care provision for MLWH through a multidisciplinary team which adopts patient-centric values [8, 15]. In the context of HIV, multidisciplinary approaches to care have been associated with numerous clinical and patient-reported advantages relative to standard of care, including higher rates of retention in care, HIV treatment adherence, and improved CD4 counts [16, 17], and have been discussed as important by MLWH in meeting their bio-psycho-social needs [15].

While previous qualitative work indicates that ART, provided rapidly and within a patient-centered multidisciplinary clinic, is well received by MLWH and seems to encourage their initial and sustained engagement with HIV care and treatment [15], quantitative evidence supporting this approach to care is lacking. More specifically, quantitatively evaluating patient-reported outcomes and experiences at several timepoints throughout the HIV care continuum, including at the early retention phase (< 6 months) and long-term retention phase (> 6 months), has been previously reported as important for studies with vulnerable populations such as MLWH [5]. Furthermore, assessing variation in patient-reported outcomes and experiences over time and by sociodemographic factors may support the identification of certain profiles of migrants that may experience more challenges and thus require more support within HIV primary care settings. The specific concepts that have been previously suggested as important in exploring for MLWH are: perceived social support, HIV-related internalized stigma, and psychological distress; treatment self-efficacy, compliance, and satisfaction; and provider empathy and cultural competence through patient-reported measures administered over the course of care engagement [8, 15]. As such, in this study, we sought to explore patient-reported outcomes and experiences on these concepts among MLWH enrolled in a multidisciplinary program with free, rapid, and onsite ART dispensation.

## Methods

### Study objectives

The objectives of this study are:

- To measure participants’ perceived social support, HIV-related internalized stigma, and psychological distress; treatment self-efficacy, compliance, and satisfaction; and provider empathy and cultural competence through patient-reported measures administered over the course of care engagement.
- To determine whether differences exist for patient-reported outcomes and experiences by sociodemographic factors and time.

### Study design & setting

In January 2020, we initiated a 96-week pilot feasibility study with a prospective cohort design (the ‘ASAP’ Study) at the Chronic Viral Illness Service of the McGill University Health Centre (CVIS/MUHC). The CVIS/MUHC is a public quaternary hospital-based clinic in Montreal, Canada, and serves the largest proportion of MLWH in the city. The CVIS/MUHC offers multidisciplinary HIV care through a team of HIV-specialist physicians, nurses, pharmacists, a social worker, a psychologist, and a psychiatrist. In this study, all participants were initiated on bictegravir/emtricitabine/tenofovir alafenamide (B/F/TAF) rapidly (i.e., within a median of 5 days) after linkage to our clinic [18]. This manuscript presents an interim analysis of patient-reported quantitative data collected from MLWH enrolled in the ASAP study up to week 48.

### Sample size

The ASAP Study’s target population consisted of new treatment-naïve PLWH at the CVIS/MUHC. Notably, the CVIS/MUHC received an average of 30 new ART-naïve MLWH annually between 2016 and 2022. For this interim analysis, 39 migrants had been enrolled in the ASAP Study since January 2020, however, 4 were either lost-to-follow-up or left the study before initiating B/F/TAF and beginning data collection. As no data were available for these migrants, analyses were completed with 35 participants. Note that all 35 participants had been enrolled in the study for at least 24 weeks and 75% had been enrolled for 48 weeks. As a non-probabilistic sampling method was used, no formal sample size calculation based on power considerations and effect sizes was done. However, it is important to note that pilot feasibly studies generally have a sample size of approximately 30 participants on average per intervention arm [18, 19]. This small sample of participants does not compromise comparisons between groups of interest. This is because a minimum of 5–10 units per group in longitudinal studies is recommended for group comparisons to assist in avoiding convergence problems which may bias parameter estimates [20–22].

### Data collection

Participants’ sociodemographic characteristics were captured at enrollment and were updated at Week 48. Sociodemographic factors include: birth region, birth year, sex, sexual orientation, living status (i.e., living alone or with others), educational level, occupational status, fluency with French (i.e., the official language of the province), health coverage, and time in Canada before being linked to the CVIS/MUHC. Data was also collected on participants’ usage of SIDEP + , which is a public integrated screening and prevention service for HIV and other sexually transmitted blood-borne infections, for conducting blood test between ASAP study visits. This is because blood tests at the CVIS/MUHC are only covered for those who have access to the provincial medicare system (RAMQ) or a collective insurance plan which covers the cost of HIV care, whereas SIDEP + provides lab tests free of charge for all residents and visitors of Quebec.

Four patient-reported outcome measures (PROMs) and four patient-reported experienced measures (PREMs) were administered at weeks 4, 24, and 48 of the study. PROMs are defined as “any report of the status of a patient’s health condition that comes directly from the patient, without interpretation of the patient’s response by a clinician or anyone else” [23]. Alternatively, PREMs provide information on “patients’ perceptions of their experience while receiving care” [24]. They concern impacts of the process of care, not its outcomes; and they indirectly inform on care *quality*, not care effectiveness. The eight previously validated patient-reported measures utilized in this study focus on three thematic areas: (1) psychosocial vulnerabilities (i.e., perceived social support, HIV-related internalized stigma, and psychological distress); (2) experience with treatment (i.e., perceived treatment self-efficacy, compliance, and satisfaction); and (3) perceptions of healthcare providers (i.e., perceived clinician cultural competence and empathy). Descriptions for the eight measures, including the way they are scored and details around their validation follow. Note that all questionnaires were administered in French, English, and/or Spanish depending on which language participants preferred. All data were housed in a data management system (i.e., REDCap). Regular quality control checks were carried out to ensure proper data input into and data export from REDCap, the last one between May 23–29, 2024.

#### Psychosocial vulnerabilities

- PROM 1 – Perceived social support was measured with the modified 8-item Medical Outcomes Study Social Support Survey (mMOS-SS) [25, 26]. For this PROM, a global score was calculated as the average score of all items, transformed to a 0 to 100 scale [26]. Higher scores suggest more perceived support. Cronbach’s alpha for the complete scale ranges from 0.88 to 0.93 [25].
- PROM 2 – Perceived HIV-related internalized stigma was measured with the 6-item Internalized AIDS-Related Stigma Scale (IA-RSS) [27]. A seventh item (“I feel uncomfortable taking my medication in front of someone else”) was added to this PROM to further explore internalized stigma. To simplify administration, the items were dichotomized (1 = Agree, 0 = Disagree). A global score was calculated as the sum of all items (range 0 to 7). Higher scores suggest more internalized stigma. Cronbach’s alpha for the original 6-item scale ranges from 0.73 to 0.76 [27].
- PROM 3 – Psychological distress in the past 30 days was examined with the 6-item Kessler Psychological Distress Scale (K6) [28]. The 5-point response scale for this PROM ranges from “None of the time” (= 1) to “All of the time” (= 5). Responses are summed to provide a global score, ranging from 6 to 30. Scores of 19 to 30 suggest “a probable serious mental illness” and scores of 6 to 18, “a probable absence of serious mental illness” [28]. A recent calculation of Cronbach’s alpha is 0.86 [29, 30].

#### Treatment self-efficacy, compliance, and satisfaction

- PROM 4 – Treatment self-efficacy (i.e., daily medication self-management) was measured with the PROMIS Self-efficacy for Managing Chronic Conditions – Managing Medications and Treatment – Short Form 4a [31]. This PROM contains 4-items answered on a 5-point Likert scale from “I am not at all confident” (= 1) to “I am very confident” (= 5). The global score is calculated by summing responses to all items (score range: 4 to 20). Higher scores suggest better self-efficacy. Cronbach’s alpha is between 0.85 and 0.92 [31].
- PREM 1 – Perceived compliance with their clinicians’ treatment plans was measured with a subscale of the Generic Medical Interview Satisfaction Scale (G-MISS) [32]. The 2 items of the compliance subscale are scored on a 6-point Likert scale from 1 to 6 (i.e., “strongly disagree” to “strongly agree”). A score is obtained for this PREM by calculating the mean of the two items and then transforming the scores into a 0 to 100 scale. Higher sores suggest greater compliance. The subscale has a Cronbach’s alpha of 0.84.
- PREM 2 – Treatment satisfaction was measured with the 10-item HIV Treatment Satisfaction Questionnaire (HIVTSQ) – status version [33]. Items for this PREM are rated from 1 to 7 (1 = least satisfied; 7 = most satisfied), with response options adjusted to the item. The sum of the 10 item scores produces the global scale score (range: 10 to 70). Higher scores suggest greater satisfaction. The measure has a Cronbach’s alpha of 0.91.

#### Perceptions of healthcare providers

- PREM 3 – Perceived provider empathy was measured with the 10-item Consultation and Relational Empathy measure (CARE) [34]. Items for this PREM are scored on a 5-point rating scale from ‘poor’ (= 1) to ‘excellent’ (= 5). The item ratings are summed to produce the global score (range: 10–50). Higher scores suggest greater perceived empathy. The measure has a Cronbach’s alpha of 0.93.
- PREM 4 – Perceived cultural competence of clinicians by patients was measured with the ‘Explaining’ subscale of the Physician Cultural Competency measure (PRPCC) [35–37]. This PREM includes 8 items which are rated on a 5-point Likert scale (1 = Never to 5 = Always). The global score is computed with the mean score for all 8-item, transformed to 0 to 100. Higher scores suggest greater perceived cultural competence. The complete scale has a Cronbach’s alpha of 0.89.

### Data analysis

All quantitative analyses were conducted using *R Statistical Software*. Means and standard deviations were calculated for each self-reported measure by timepoint and sociodemographic factor. Note that time was treated as a continuous variable in this study. Following descriptive analyses, linear mixed models were fitted to the data [38]. Linear mixed models are well suited for analyzing longitudinal data with small sample sizes [39]. Sociodemographic characteristics at enrollment were used for analyses at Weeks 4 and 24, and the updated sociodemographic characteristics were used for analysis at Week 48. The following characteristics were considered to have a fixed effect (i.e., these variables have a constant and consistent influence on the patient-reported measures for all individuals within a particular group): birth region, birth year, sex, sexual orientation, and time in Canada before first visit to the CVIS/MUHC. The other characteristics (i.e., living status, education level, occupational status, fluency with French, health coverage, and SIDEP + usage for blood tests) were considered to have a mixed effect (i.e., these variables may have both a constant and varying influence on the outcome across individuals within groups). To identify the most appropriate analytical model, the Maximum Likelihood Estimation approach was utilized [21, 40–42]. The model with the lowest Akaike Information Criterion score for each self-reported measure was chosen and subjected to the Restricted Maximum Likelihood (REML) approach [21, 40–42]. To reduce bias introduced by the non-probabilistic sampling method and to enhance generalizability of the results, REML parameters were estimated using a non-parametric bootstrap resampling approach for computing p-values [43–45]. The bootstrap method is particularly useful when the sample size is insufficient for accurate statistical inference or when selection bias is a concern [21, 43–45]. Specifically, we bootstrapped 10,000 samples. Bootstrapped p-values are reported, with a significance level set at < 0.05.

### Patient and stakeholder engagement

This study is grounded in patient-oriented research which focuses on: engaging patients and relevant stakeholders as partners, responding to patient-identified priorities, and ultimately improving patient outcomes [46]. During the ASAP Study, an advisory committee (the ASAP Migrant Advisory Committee), was developed [8, 14, 15]. Members of the ASAP Migrant Advisory Committee contributed to the revision and editing of this manuscript.

### Ethics

This study was conducted in accordance with applicable Health Canada regulations, International Conference on Harmonisation guidelines on current Good Clinical Practice, and the Declaration of Helsinki. It was approved by the Research Ethics Board of the Research Institute of the McGill University Health Centre (reference #: MP-37-2020-4911).

## Results

### Sociodemographic characteristics

At enrollment, more than half of the participants: came from Africa and/or the Caribbean (n = 20, 57%); were 35 or older (n = 20, 57%); were male (n = 28, 80%); identified as gay, lesbian, or bisexual with respect to their sexual orientation (n = 22, 63%); lived with others (n = 27, 77%); had university-level education (n = 20, 57%); were unemployed (n = 24, 69%); did not speak French (n = 20, 57%); had sufficient health coverage for HIV-related needs through public health insurance (n = 20, 57%); used SIDEP + for at least one blood test (n = 13, 37%); and spent less than 1 year in Canada before being linked to the CVIS/MUHC (n = 20, 57%). These values remained relatively consistent at Week 48. Descriptive statistics by sociodemographic factor at enrolment and at Week 48 are provided in Table 1.

**Table 1:** Participant characteristics by study week

	Enrolment	Week 48
	n = 35	n = 26
Birth region		
African, Caribbean	20 (57%)	17 (65%)
Other	15 (43%)	9 (35%)
Age		
Less than 35	15 (43%)	13 (50%)
35 or more	20 (57%)	13 (50%)
Sex		
Female	7 (20%)	6 (23%)
Male	28 (80%)	20 (77%)
Sexual orientation		
Heterosexual	13 (37%)	10 (38%)
Lesbian, gay, bisexual	22 (63%)	16 (62%)
Living status		
Alone	7 (20%)	9 (35%)
With others	27 (77%)	17 (65%)
Not reported	1 (3%)	–
Educational level		
Less than university	15 (43%)	12 (46%)
University	20 (57%)	14 (54%)
Occupational status		
Unemployed	24 (69%)	9 (35%)
Paid employment or student	11 (31%)	17 (65%)
French fluency		
No	20 (57%)	14 (54%)
Yes	15 (43%)	12 (46%)
Health coverage		
Private or none	15 (43%)	10 (38%)
Public	20 (57%)	16 (62%)
Used SIDEP + for at least one Blood Test		
No	22 (63%)	20 (77%)
Yes	13 (37%)	6 (23%)
Time from arriving in Canada to first visit at the CVIS/MUHC		
Less than 1 year	20 (57%)	16 (62%)
1 year or more	13 (37%)	9 (35%)
Not reported	2 (6%)	1 (4%)

### Psychosocial vulnerabilities

#### Social support

The mMOS-SS mean scores (and standard deviations) for the entire sample were 59.4 (26.5), 65.5 (26.5), and 52.6 (30.4) at weeks 4, 24, and 48 respectively (Table 2). These scores suggest that on average, throughout the 48 weeks, participants perceived having moderate levels of social support. Significant differences were identified by birth region, where those from Africa and/or the Caribbeans perceived having less social support compared to people from other regions (p = 0.03). No significant differences were identified by the remaining sociodemographic characteristics or time (Table 3).

**Table 2:** Mean scores (with standard deviations) by week and sociodemographic characteristics for self-reported measures associated with psychosocial vulnerabilities

	MOS-SSS			IA-IRSS			K6		
	Week 4	Week 24	Week 48	Week 4	Week 24	Week 48	Week 4	Week 24	Week 48
All participants	59.4 (26.5)	65.5 (26.5)	54.1 (30.6)	4.43 (2.0)	3.91 (2.3)	4.08 (2.3)	12.5 (5.2)	11.4 (5.1)	11.5 (5.3)
Birth region									
African and/or Caribbean	52.2 (24.0)	55.9 (24.9)	43.6 (26.5)	4.71 (1.7)	4.35 (2.3)	4.38 (2.1)	11.9 (4.4)	10.9 (4.2)	11.6 (5.8)
Other	68.1 (27.5)	54.8 (28.3)	72.9 (29.5)	4.08 (2.4)	3.40 (2.3)	3.56 (2.8)	13.3 (6.3)	11.9 (6.1)	11.2 (4.7)
Age									
Less than 35	69.5 (24.1)	66.3 (26.7)	54.8 (31.8)	4.43 (2.2)	4.54 (2.2)	4.38 (2.5)	11.4 (6.1)	11.1 (7.0)	10.5 (5.6)
35 or more	52.1 (26.3)	48.3 (23.6)	53.4 (30.6)	4.44 (2.0)	3.47 (2.4)	3.75 (2.2)	13.3 (4.5)	11.5 (3.4)	12.5 (5.0)
Sex									
Female	73.4 (23.9)	44.8 (27.7)	49.0 (33.1)	4.00 (0.7)	4.50 (1.4)	4.17 (2.0)	9.00 (2.7)	10.3 (4.4)	9.67 (3.0)
Male	56.0 (26.4)	57.8 (25.6)	55.8 (30.5)	4.54 (2.3)	3.77 (2.5)	4.05 (2.5)	13.3 (5.4)	11.6 (5.3)	12.0 (5.8)
Sexual orientation									
Heterosexual	70.7 (23.2)	54.8 (27.8)	50.7 (33.0)	4.36 (1.3)	4.45 (1.8)	4.22 (1.9)	9.50 (2.5)	9.92 (4.0)	10.2 (5.3)
Lesbian, gay, bisexual	53.1 (26.6)	55.7 (25.8)	56.1 (30.1)	4.47 (2.4)	3.62 (2.6)	4.00 (2.6)	14.4 (5.7)	12.1 (5.5)	12.3 (5.4)
Living status									
Alone	43.3 (19.6)	56.3 (27.9)	61.1 (24.0)	5.50 (1.6)	4.29 (2.6)	3.89 (2.6)	17.4 (6.4)	13.6 (7.5)	11.4 (4.6)
With others	63.0 (26.8)	56.9 (25.1)	50.2 (33.8)	4.22 (2.1)	3.79 (2.4)	4.19 (2.3)	11.1 (4.1)	10.5 (4.1)	11.5 (5.8)
Educational level									
Less than university	66.7 (24.5)	58.4 (29.0)	63.5 (28.9)	4.77 (1.5)	4.00 (2.2)	4.17 (2.2)	11.0 (3.9)	10.4 (4.6)	10.9 (4.3)
University	54.8 (27.2)	53.4 (24.5)	45.4 (30.5)	4.18 (2.4)	3.84 (2.5)	4.00 (2.6)	13.6 (5.9)	12.0 (5.4)	11.9 (6.2)
Occupational status									
Unemployed	60.0 (27.6)	62.9 (23.9)	56.3 (30.1)	4.10 (2.1)	3.67 (2.2)	4.56 (2.4)	11.6 (5.5)	11.3 (5.7)	11.0 (5.1)
Paid employment or Student	58.2 (25.6)	40.3 (24.4)	52.9 (31.7)	5.10 (1.9)	4.36 (2.5)	3.81 (2.4)	14.3 (4.4)	11.4 (3.8)	11.7 (5.6)
French fluency									
No	61.8 (25.7)	45.2 (23.6)	51.8 (32.5)	4.67 (1.9)	3.95 (2.5)	4.43 (2.5)	13.5 (5.9)	12.4 (5.5)	11.5 (4.5)
Yes	56.5 (28.0)	69.2 (23.3)	57.1 (29.2)	4.08 (2.3)	3.85 (2.2)	3.64 (2.2)	11.1 (3.9)	10.0 (4.3)	11.4 (6.4)
Health coverage									
Private or none	52.9 (27.6)	54.5 (29.5)	62.5 (34.0)	4.57 (2.1)	3.86 (2.4)	3.80 (2.4)	12.3 (4.8)	10.1 (3.8)	11.5 (5.2)
Public	65.4 (24.7)	56.1 (24.0)	48.5 (27.8)	4.31 (2.0)	3.94 (2.4)	4.27 (2.3)	12.8 (5.7)	12.4 (5.8)	11.4 (5.6)
Used SIDEP + for at least one blood test since last appointment									
No	60.2 (26.6)	53.3 (24.9)	56.3 (29.5)	3.83 (2.2)	3.58 (2.6)	3.89 (2.5)	11.8 (4.4)	11.1 (4.4)	10.7 (5.3)
Yes	58.2 (27.3)	58.7 (28.4)	47.4 (35.9)	5.33 (1.5)	4.38 (1.9)	4.67 (2.1)	13.6 (6.3)	11.7 (6.2)	14.0 (5.0)
Time in Canada before first CVIS/MUHC visit									
Less than 1 year	62.9 (23.4)	63.7 (24.2)	54.8 (29.5)	4.07 (1.9)	3.81 (2.4)	3.33 (2.1)	10.5 (4.2)	10.4 (4.2)	11.3 (4.5)
1 year or more	52.3 (30.0)	53.8 (26.3)	48.6 (32.4)	5.17 (1.9)	4.08 (2.3)	5.78 (1.5)	14.3 (5.8)	11.7 (6.3)	12.4 (6.7)

**Table 3:** Boot-strapped p-values using the REML approach for all self-reported measures

	MOS-SSS	IA-IRSS	K6	PROMIS	G-MISS	HIVTSQ	CARE	PRPCC
Week	0.31	0.72	0.92	0.93	0.084	0.69	0.32	0.25
Birthreg (Other)	**0.03**	**0.002**	–	–	–	**0.0008**	–	–
EnrolmentAge (Lessthan35)	–	**0.0007**	–	–	–	**0.0057**	–	–
Sex (Male)	–	0.17	–	–	–	0.12	–	–
SexuOrien (LGB)	–	0.86	**0.0021**	–	–	0.24	–	–
Educationlevel (University)	–	0.71	–	–	–	0.32	–	0.24
HealthCov (Public)	–	0.81	–	–	–	0.063	–	–
Occupationalstat (Unemployed)	–	0.25	–	–	0.10	0.77	–	–
Livingstat (With others)	–	0.86	–	–	–	0.22	–	–
FrenchFluency (Yes)	–	**0.0033**	–	–	–	0.82	–	–
BloodtestatSIDEP (Yes)	–	0.15	–	–	–	0.092	0.38	–
TimeinCADbefore 1stCVISVisit (Lessthan1year)	0.14	0.07	0.11	0.053	0.28	0.12	0.28	0.44

‘- ‘ means that the factor was not identified as being part of the chosen model using the Maximum Likelihood approach and is therefore considered insignificant

Bold text indicates a significant p-value

#### Internalized HIV-related stigma

The IA-RSS mean scores (and standard deviations) for the entire sample were 4.4 (2.0), 3.9 (2.3), and 4.1 (2.3) at weeks 4, 24, and 48 respectively (Table 2). These scores suggest that on average, throughout the 48-weeks, participants experienced elevated levels of internalized HIV-related stigma. Significant differences were identified by: birth region, where those from Africa and/or the Caribbeans perceived higher levels of internalized stigma compared to people from other regions (p = 0.002); age, where those less than 35 perceived higher levels of internalized stigma compared to those 35 and older (p = 0.0007); and French fluency, where those not fluent in French perceived higher levels of internalized stigma compared to those fluent in French (p = 0.0033). No significant differences were identified by the remaining sociodemographic characteristics and time (Table 3).

#### Psychological distress

The K6 mean scores (and standard deviations) for the entire sample were 12.5 (5.2), 11.4 (5.1), and 11.5 (5.3) at weeks 4, 24, and 48 respectively (Table 2). These scores suggest that on average, throughout the 48 weeks, participants did not experience levels of psychological distress that were indicative of a serious mental illness. Significant differences were identified by sexual orientation, where those who identified as gay or bisexual experienced higher distress compared to those who identified as heterosexual (p = 0.0021). No significant differences were identified by the remaining sociodemographic characteristics or time (Table 3).

### Treatment self-efficacy, compliance, and satisfaction

#### Treatment-self-efficacy

The PROMIS Self-efficacy mean scores (and standard deviations) for the entire sample were 16.7 (4.2), 17.3 (3.5), and 16.5 (3.7) at weeks 4, 24, and 48 respectively (Table 4). These scores suggest that on average, throughout the 48 weeks, participants felt high self-efficacy with respect to daily medication self-management. No significant differences were identified by sociodemographic characteristics or time (Table 3).

**Table 4:** Mean scores (with standard deviations) by week and sociodemographic characteristics for self-reported measures associated with treatment adherence and satisfaction

	PROMIS			G-MISS			HIVTSQ		
	Week 4	Week 24	Week 48	Week 4	Week 24	Week 48	Week 4	Week 24	Week 48
All participants	16.7 (4.2)	17.3 (3.5)	16.5 (3.7)	88.4 (23.0)	77.6 (28.4)	81.6 (23.6)	62.3 (6.5)	63.9 (6.1)	61.3 (6.8)
Birth region									
African and/or Caribbean	16.4 (4.7)	16.6 (3.9)	15.8 (4.0)	92.2 (13.5)	71.1 (32.8)	80.6 (25.4)	61.5 (7.5)	62.3 (7.2)	59.5 (7.5)
Other	17.3 (3.4)	18.3 (2.8)	17.9 (2.6)	83.6 (31.3)	86.0 (19.6)	83.3 (21.2)	63.4 (5.0)	66.0 (3.3)	64.8 (3.7)
Age									
Less than 35	16.0 (4.6)	17.3 (2.6)	15.2 (4.4)	92.0 (17.8)	65.7 (36.7)	4.2 (23.9)	61.8 (7.2)	62.4 (7.3)	60.0 (6.7)
35 or more	17.3 (3.9)	17.4 (4.1)	17.8 (2.3)	85.3 (27.0)	86.0 (17.3)	88.5 (21.9)	62.6 (6.1)	65.0 (4.9)	62.7 (6.9)
Sex									
Female	17.4 (2.7)	16.3 (4.0)	14.8 (4.8)	100 (0)	68.6 (34.8)	85.0 (17.6)	59.3 (8.8)	64.4 (5.4)	61.3 (6.1)
Male	16.6 (4.5)	17.6 (3.4)	17.0 (3.2)	85.2 (25.2)	80.0 (26.7)	80.5 (25.5)	63.1 (5.7)	63.8 (6.3)	61.4 (7.2)
Sexual orientation									
Heterosexual	16.9 (4.4)	16.8 (3.4)	15.1 (4.7)	95.8 (14.4)	71.7 (36.9)	86.7 (16.6)	61.4 (7.1)	63.9 (5.3)	59.9 (5.9)
Lesbian, gay, bisexual	16.6 (4.2)	17.6 (3.6)	17.4 (2.7)	84.0 (26.2)	80.9 (22.9)	78.8 (26.8)	62.8 (6.3)	63.9 (6.5)	62.3 (7.4)
Living status									
Alone	15.6 (3.8)	18.7 (0.8)	18.1 (2.4)	75.7 (23.0)	80.0 (16.3)	83.3 (25.0)	59.3 (6.4)	63.1 (6.6)	62.3 (8.1)
With others	17.2 (4.3)	17.2 (3.7)	15.6 (4.0)	91.7 (22.6)	76.9 (31.6)	80.6 (23.5)	63.4 (6.4)	64.1 (6.1)	60.8 (6.3)
Educational level									
Less than university	16.3 (4.0)	17.3 (2.4)	15.0 (3.9)	93.8 (17.1)	72.1 (30.4)	85.8 (14.4)	62.1 (6.7)	62.1 (7.1)	60.4 (5.3)
University	17.1 (4.4)	17.4 (4.2)	17.8 (3.0)	84.7 (26.1)	81.5 (27.0)	77.7 (29.8)	62.4 (6.5)	65.2 (5.0)	62.1 (8.0)
Occupational status									
Unemployed	17.0 (4.0)	17.7 (2.4)	14.6 (3.8)	88.6 (25.6)	71.3 (32.2)	70.0 (17.7)	62.6 (6.3)	63.9 (6.2)	59.2 (4.6)
Paid employment or student	16.1 (4.7)	16.5 (5.2)	17.5 (3.3)	88.2 (18.3)	90.9 (9.4)	87.1 (24.4)	61.6 (7.2)	64.0 (6.1)	62.5 (7.6)
French fluency									
No	16.5 (4.3)	17.4 (4.1)	17.9 (2.3)	85.0 (27.9)	78.9 (25.8)	81.4 (25.1)	63.0 (6.1)	64.3 (5.0)	62.1 (6.5)
Yes	17.0 (4.2)	17.3 (2.6)	14.8 (4.3)	92.9 (14.4)	76.0 (32.2)	81.8 (22.7)	61.4 (7.1)	63.5 (7.4)	60.4 (7.3)
Health coverage									
Private or none	17.2 (4.2)	18.4 (2.0)	17.6 (2.9)	87.3 (25.8)	90.7 (11.0)	90.0 (11.5)	62.2 (6.0)	64.9 (5.0)	64.4 (4.8)
Public	16.4 (4.3)	16.5 (4.2)	15.8 (4.0)	89.4 (21.1)	67.4 (33.6)	76.0 (28.0)	62.3 (7.0)	63.1 (6.8)	59.4 (7.3)
Used SIDEP + for at least one blood test since last appointment									
No	17.1 (3.8)	16.8 (4.1)	15.9 (3.8)	94.2 (13.0)	74.3 (33.1)	78.9 (26.0)	63.5 (6.4)	64.1 (6.7)	60.7 (7.2)
Yes	16.1 (4.8)	18.2 (2.2)	18.5 (2.5)	80.0 (31.4)	83.1 (18.4)	90.0 (11.0)	60.4 (6.5)	63.6 (5.2)	63.7 (5.2)
Time in Canada before first CVIS/MUHC visit									
Less than 1 year	15.6 (5.0)	16.5 (4.1)	15.8 (3.7)	95 (14.0)	76.5 (30.0)	81.3 (22.6)	63.4 (6.5)	62.2 (7.2)	60.4 (6.8)
1 year or more	17.8 (3.3)	18.9 (1.4)	17.4 (3.6)	80 (30.1)	76.9 (31.2)	80.0 (26.9)	60.9 (6.8)	66.5 (4.1)	62.1 (6.9)

#### Compliance

The G-MISS compliance subscale mean scores (and standard deviations) for the entire sample were 88.4 (23.0), 77.6 (28.4), and 81.6 (23.6) at weeks 4, 24, and 48 respectively (Table 4). These scores suggest that on average, throughout the 48 weeks, participants perceived great compliance with their clinicians’ treatment plans. No significant differences were identified by sociodemographic characteristics or time (Table 3).

#### Treatment satisfaction

The HIVTSQ mean scores (and standard deviations) for the entire sample were 62.3 (6.5), 63.9 (6.1), and 61.3 (6.8) at weeks 4, 24, and 48 respectively (Table 4). These scores suggest that on average, throughout the 48 weeks, participants felt high satisfaction with their treatment. Significant differences were identified by: birth region, where those from Africa and/or the Caribbeans had lower treatment satisfaction compared to those from other regions (p = 0.0008); and age, where those less than 35 had lower treatment satisfaction compared to those 35 and older (p = 0.0057). No significant differences were identified by the remaining sociodemographic characteristics and time (Table 3).

### Perceptions around healthcare providers

#### Provider empathy

The CARE mean scores (and standard deviations) for the entire sample were 45.1 (6.1), 46.5 (7.0), and 47.5 (3.8) at weeks 4, 24, and 48 respectively (Table 5). These scores suggest that on average, throughout the 48 weeks, participants perceived high levels of empathy from their healthcare providers. No significant differences were identified by sociodemographic characteristics or time (Table 3).

**Table 5:** Mean scores (with standard deviations) by week and sociodemographic characteristics for self-reported measures associated with perceptions around heath care providers

	CARE			PRPCC		
	Week 4	Week 24	Week 48	Week 4	Week 24	Week 48
All participants	45.1 (6.1)	46.5 (7.0)	47.5 (3.8)	92.0 (10.5)	93.3 (9.9)	89.1 (14.7)
Birth region						
African and/or Caribbean	45.7 (6.5)	46.0 (7.5)	47.5 (3.9)	90.3 (11.7)	89.8 (12.2)	87.7 (16.5)
Other	44.3 (5.5)	47.1 (6.5)	47.5 (3.9)	94.2 (8.7)	96.9 (5.3)	91.3 (11.8)
Age						
Less than 35	46.0 (5.6)	46.9 (5.6)	47.6 (4.0)	92.3 (10.8)	92.4 (11.3)	84.4 (17.3)
35 or more	44.4 (6.5)	46.2 (7.9)	47.3 (3.8)	91.7 (10.6)	93.9 (9.2)	93.8 (10.3)
Sex						
Female	43.2 (6.5)	44.8 (7.0)	47.2 (3.9)	89.3 (12.7)	90.6 (11.7)	81.9 (17.7)
Male	45.7 (6.0)	46.8 (7.1)	47.6 (3.9)	92.8 (10.0)	93.9 (9.7)	91.0 (13.8)
Sexual orientation						
Heterosexual	44.7 (5.8)	46.9 (5.6)	48.4 (3.1)	91.7 (11.9)	92.5 (11.6)	85.1 (18.7)
Lesbian, gay, bisexual	45.4 (6.4)	46.2 (7.8)	46.9 (4.2)	92.2 (9.9)	93.8 (9.3)	91.5 (11.8)
Living status						
Alone	44.8 (6.5)	45.2 (7.3)	47.1 (4.6)	90.2 (9.6)	95.5 (7.2)	94.1 (10.1)
With others	45.5 (6.1)	47.3 (6.6)	47.7 (3.3)	93.2 (10.5)	92.6 (10.9)	86.5 (16.3)
Educational level						
Less than university	43.9 (6.6)	46.5 (6.3)	46.6 (4.3)	93.0 (10.2)	91.1 (11.8)	80.7 (15.8)
University	45.9 (5.7)	46.4 (7.7)	48.3 (3.2)	91.3 (11.0)	94.8 (8.6)	96.2 (9.5)
Occupational status						
Unemployed	46.8 (5.0)	47.4 (5.1)	47.5 (4.4)	92.9 (10.3)	92.5 (11.3)	81.3 (15.9)
Paid employment or Student	42.3 (7.0)	44.5 (10.0)	47.5 (3.6)	90.3 (11.2)	95.0 (6.6)	93.0 (12.9)
French fluency						
No	46.0 (5.2)	46.6 (6.4)	46.4 (4.1)	94.1 (8.7)	94.9 (7.4)	91.1 (11.7)
Yes	43.8 (7.2)	46.3 (8.0)	49.1 (2.7)	89.3 (12.3)	90.6 (13.3)	86.3 (18.5)
Health coverage						
Private or none	45.2 (5.9)	45.2 (9.0)	46.6 (4.0)	91.0 (11.4)	95.6 (8.4)	91.3 (11.6)
Public	45.0 (6.4)	47.3 (5.3)	47.9 (3.7)	92.8 (9.9)	91.8 (10.8)	87.7 (16.6)
Used SIDEP + for at least one blood test since last appointment						
No	46.7 (4.2)	47.5 (5.1)	47.9 (3.6)	93.3 (10.4)	93.3 (9.6)	88.8 (15.6)
Yes	43.2 (7.5)	44.6 (9.7)	45.8 (4.6)	90.1 (10.8)	93.5 (11.0)	90.0 (12.4)
Time in Canada before first CVIS/MUHC visit						
Less than 1 year	45.8 (5.5)	44.1 (9.4)	47.6 (3.7)	93.3 (10.8)	91.6 (11.4)	86.5 (16.0)
1 year or more	44.3 (7.1)	48.3 (4.2)	47.2 (4.2)	91.5 (10.8)	95.9 (9.0)	93.3 (11.5)

#### Provider cultural competence

The PRPCC explaining subscale mean scores (and standard deviations) for the entire sample were 92.0 (10.5), 93.3 (9.9), and 89.1 (14.7) at weeks 4, 24, and 48 respectively (Table 5). These scores suggest that on average, throughout the 48 weeks, participants perceived high levels of cultural competence from their clinicians. No significant differences were identified by sociodemographic characteristics or time (Table 3).

## Discussion

This study explores the patient-reported outcomes and experiences of MLWH enrolled in a prospective cohort study in Montreal, Canada, where B/F/TAF was being dispensed free-of-charge, onsite, and rapidly after linkage to multidisciplinary HIV care. Specifically, across weeks 4, 24, and 48, self-reported measures were used to assess perceived social support, internalized HIV-related stigma, and psychological distress; treatment compliance, self-efficacy, and satisfaction; and participant perceptions around their healthcare providers’ cultural competence and empathy. To our knowledge, this is the first study that provides quantitative insights on these concepts through self-reported measures among MLWH enrolled in such a model of primary HIV care.

### Psychosocial vulnerabilities

Throughout follow-up, there was a low probability that MLWH experienced a serious mental illness based on their K6 psychological distress scores. However, those who identified as gay or bisexual experienced higher levels of distress. Furthermore, MLWH expressed elevated levels of internalized HIV-related stigma and moderate levels of social support. Notably, those who were from Africa or the Caribbean perceived having less social support. Additionally, those from Africa or the Caribbean, those who were less than 35, and those who were not fluent in French experienced a significantly higher degree of internalized stigma. To some extent, these findings are consistent with those of other studies conducted in Canada and other regions among MLWH, PLWH, and general populations of international migrants [47–56]. It is well recognized that migrants often experience higher levels of stigma, mental illness, and challenges with accessing, building, and maintaining social support [51]. The further burden of living with HIV can amplify these challenges [8, 15, 50, 52, 53]. For example, whereas migrants in general struggle with obtaining legal status in their new country, newly-diagnosed MLWH express heightened concern around deportation as a result of stigma, discrimination, and fear from their positive diagnosis [8, 15]. Moreover, when additional intersectional burdens are experienced, such as discrimination and stigma due to skin colour or race, or when self-perceived limitations are identified (e.g., lack of ability to speak proficiently in the host nation’s language), levels of internalized stigma among PLWH can increase [8, 54]. Also, several studies have previously noted that younger PLWH may experience higher levels of stigma compared to older PLWH [50, 55, 56]. The relationship between age and stigma among MLWH may be attributed to the different life-stages people occupy (e.g., international student versus an established professional), the social networks people have established at different ages, and the coping mechanisms that people have developed and strengthened over time [50]. While engaged in this cohort study, psychosocial vulnerabilities were not found to decrease significantly over time. This may be linked to the idea that the first year of moving to a new country, learning about one’s HIV diagnosis, and engaging in care and treatment, can be a very challenging time across the emotional, mental, and social levels for individuals [15, 57, 58]. Additionally, perhaps more time (e.g., over 1–2 years) is needed to see a change in these aspects of participants’ lives. Though self-reported measures, like those used in this study, may be helpful in identifying patient perspectives, long time lags are noted to exist for health effects to manifest when dealing with changes to upstream social determinants of health [59].

### Treatment self-efficacy, compliance, and satisfaction

Throughout the 48-weeks, participants reported a high degree of treatment self-efficacy, compliance, and satisfaction. Given that some scholars suggest that treatment self-efficacy can be significantly affected by mental health challenges, this finding is unique and important [60]. In previous qualitative work with MLWH enrolled in the ‘ASAP’ study, MLWH expressed high satisfaction with B/F/TAF, and noted the importance of feeling control over their HIV, as well as a strong sense of responsibility for managing their HIV [15]. Alongside these individual characteristics, the adoption of person-centered approaches to care may be central to enabling a high degree of daily medication self-management [15]. Importantly, while levels of treatment self-efficacy, compliance, and satisfaction where high overall in this study, it was also found that those born in Africa and/or the Caribbean and those who were less than 35 years of age had a significantly lower level of satisfaction. The relationship between social factors and treatment satisfaction is complex and not well explored, particularly in the context of HIV among migrant populations. One study describes that racial and ethnic differences in satisfaction may occur based on differences in attitudes and expectations, particularly around patients’ trust with medical care systems [61]. Another study suggests that older patients may be more satisfied with their healthcare potentially due to generational factors (e.g., those raised during certain periods, such as the early days of the HIV pandemic, may be more experienced with significant hardships, and thus more accepting of inadequacies in healthcare systems) [62]. Furthermore, findings in this study may be associated with the intersectional challenges that affect these sub-populations’ psychosocial vulnerabilities [8], but a thorough qualitative exploration is warranted to better understand these phenomena.

### Perceptions around healthcare providers

Throughout the 48-week period, MLWH perceived high empathy and cultural competence from their clinical team at the CVIS/MUHC. MLWH encompass a diverse group of people, from different ethnicities, cultures, and regions. Despite this, no significant difference was identified by birth region or any other sociodemographic factor with respect to these variables. This finding is interesting given that previous studies have reported poorer satisfaction with healthcare services among migrant populations compared to native-born populations [63, 64]. Perhaps this reflects the CVIS/MUHC staff’s experience with working with MLWH. Indeed, these results validate earlier qualitative findings in which MLWH that were receiving care at the CVIS/MUHC discussed their experience of humanizing clinical encounters [15]. These encounters were characterized by feelings of kindness, acceptance, respect, safety, and trust from and with their clinicians [15]. Literature suggests that when clinicians adopt such qualities with their patients, they can help promote better rapport-building, higher quality of care, and higher levels of medication self-efficacy [15, 65–67]. Additionally, these humanizing qualities are considered essential to develop and sustain people-centered health systems [68]. However, despite rating their clinical team highly in empathy and cultural competence, MLWH’s psychosocial challenges persisted to some extent across the analytical period. This suggests that humanizing care must be coupled with specific interventions to thoroughly understand and address the complex psychosocial challenges MLWH present with.

### Strengths and limitations

A major limitation in this study is the small sample size. As this study was initiated in Jan 2020, a large portion of study recruitment took place during the COVID-19 pandemic. As a result of the limited sample, migrants originating from Africa and the Caribbean were grouped together in the linear mixed modelling analysis. Though previous work in the HIV field has grouped African, Caribbean, and Black populations based on ethnicity and other intersectional challenges experienced by these groups, it is important to acknowledge that Africa and the Caribbean are geographically separate areas of the world, and there can often be large heterogeneity within populations coming from these regions. Another limitation in this study is the use of interim data (i.e., the analysis presented in this study pertains to the halfway point of the 96 week-long ASAP cohort study). However, interim analyses in longitudinal clinical studies, as presented here, are reliable and rational approaches to report findings without comprising validity or integrity [69]. Such analyses are important for making data and summarized findings available to target audiences in a timely manner, as well as guiding the potential termination or appropriate modifications in sample size or study design [69]. Notably, by engaging in this process at the mid-point of the *ASAP* study, we were able to ensure the quality and rigor of our data collection and analysis. Another potential limitation in this study is that the IA-RSS scale was modified by adding one item to further explore internalized stigma. Though we have detailed the item we added in the methods section, and its relevance is warranted; its addition does affect the validity of the IA-RSS scale. Given that the objectives of this study were to measure participants’ self-reported outcomes and experiences, and explore differences by sociodemographic factors and time, the actual utilization of healthcare services (e.g., the number of times patients accessed social worker services) were not examined. Additionally, only a small number of female MLWH agreed to join this cohort study. This is a frequently encountered challenge in HIV clinical research [70] and has been previously reported by our team [15]. However, linear mixed modelling analysis is well suited for small samples, and bootstrapping further helps attenuate the small sample size’s effect. Furthermore, the repeated measurement approach (i.e., conducting evaluations at week 4, 24, and 48) and consideration of changes in sociodemographic factors at week 48 allowed for a more rigorous data analysis.

## Conclusions

To our knowledge, this is the first study that longitudinally explores the perspectives of MLWH around their perceived social support, internalized HIV-related stigma, and psychological distress; treatment compliance, self-efficacy, and satisfaction; and participant perceptions around their healthcare providers’ cultural competence and empathy. Importantly, findings suggest that most MLWH enrolled in this study expressed high self-efficacy, compliance, and satisfaction with their treatment, and concurrently perceived high cultural competency and empathy from their clinical care providers. Perceived social support, internalized stigma, and distress, however, could be improved among MLWH. In this regard, special attention should be given to people originating from Africa and/or the Caribbean, those less than 35, those not fluent in the native language of their host province, and those identifying as gay or bisexual. These findings potentially underscore the need to embed targeted, well-funded, and accessible mental health support within HIV care models, and that further research is required to better understand how to meet the complex and multifaceted psychosocial needs of MLWH in clinical settings.

## Data Availability

Data can be accessed upon reasonable request by contacting the corresponding author.
